# Distinguishing bipolar and major depressive disorders by brain structural morphometry: a pilot study

**DOI:** 10.1186/s12888-015-0685-5

**Published:** 2015-11-21

**Authors:** Germaine Fung, Yi Deng, Qing Zhao, Zhi Li, Miao Qu, Ke Li, Ya-wei Zeng, Zhen Jin, Yan-tao Ma, Xin Yu, Zhi-ren Wang, David H. K. Shum, Raymond C. K. Chan

**Affiliations:** 1 Neuropsychology and Applied Cognitive Neuroscience Laboratory, Key Laboratory of Mental Health, Institute of Psychology, Chinese Academy of Sciences, Beijing, China; 2 Department of Psychology, the Chinese University of Hong Kong, Hong Kong Special Administrative Region, China; 3 Cognitive Analysis & Brain Imaging Laboratory, MIND Institute, University of California, Davis, USA; 4 School of Applied Psychology and Behavioral Basis of Health Program, Griffith Health Institute, Griffith University, Brisbane, Australia; 5 University of Chinese Academy of Sciences, Beijing, China; 6 Department of Encephalopathy, Beijing University of Chinese Medicine the 3rd Affiliated Hospital, Beijing, China; 7 MRI Center of Beijing 306 Hospital, Beijing, China; 8 Peking University Sixth Hospital, Beijing, China; 9 Peking University Institute of Mental Health, Beijing, China; 10 Key Laboratory of Mental Health, Ministry of Health (Peking University), Beijing, China; 11 Center for Biological Psychiatry, Beijing Hui-Long-Guan Hospital, Beijing, China; 12 Menzies Health Institute Queensland and School of Applied Psychology, Griffith University, Gold Coast, Australia; 13 Institute of Psychology, Chinese Academy of Sciences, 16 Lincui Road, Beijing, 100101 China

**Keywords:** Bipolar disorder, Depression, Cortical Thickness, Surface area, Support vector machine

## Abstract

**Background:**

The clinical presentation of common symptoms during depressive episodes in bipolar disorder (BD) and major depressive disorder (MDD) poses challenges for accurate diagnosis. Disorder-specific neuroanatomical features may aid the development of reliable discrimination between these two clinical conditions.

**Methods:**

For our sample of 16 BD patients, 19 MDD patients and 29 healthy volunteers, we adopted vertex-wise cortical based brain imaging techniques to examine cortical thickness and surface area, two components of cortical volume with distinct genetic determinants. Based on specific characteristics of neuroanatomical features, we then used support vector machine (SVM) algorithm to discriminate between patients with BD and MDD.

**Results:**

Compared to MDD patients, BD patients showed significantly larger cortical surface area in the left bankssts, precuneus, precentral, inferior parietal, superior parietal and the right middle temporal gyri. In addition, larger volumes of subcortical regions were found in BD patients. In SVM discriminative analyses, the overall accuracy was 74.3 %, with a sensitivity of 62.5 % and a specificity of 84.2 % (*p* = 0.028). Compared to controls, larger surface area in the temporo-parietal regions were observed in BD patients, and thinner cortices in fronto-temporal regions were observed in MDD patients, especially in the medial orbito-frontal area.

**Conclusions:**

These findings have demonstrated distinct spatially distributed variations in cortical thickness and surface area in patients with BD and MDD, suggesting potentially varying etiological and neuropathological processes in these two conditions. The employment of multimodal classification on disorder-specific biological features has shed light to the development of potential classification tools that could aid diagnostic decisions.

## Background

Whether bipolar disorder (BD) and major depressive disorder (MDD) are two discrete diagnostic entities or they belong to the same disorder spectrum has received considerable research interest during the past decade. These two psychiatric conditions exhibit similar severe depressive symptoms, but show no difference in the duration of affective episodes during the course of illness [1]. However, in clinical practice, reliably distinguishing between BD and MDD is paramount for clinicians to avoid risks of misdiagnosis and inappropriate medication treatments. Although BD consists of alternating episodes of depression and mania/hypomania, the early presentation of depressive symptoms in these patients often increases the chances of them being misdiagnosed as MDD during the early onset stage. In addition, clinical studies reported higher prevalence of depressive relative to manic/hypomanic symptoms in BD. The presence of subthreshold manic symptoms during a depressive episode also made the distinction between BD and MDD difficult [2]. Thus, it poses a major challenge for clinicians to reliably diagnose BD and MDD, particularly when recurring episodes of depression are the primary common affective symptoms in both conditions [3].

Emerging evidence suggests that BD and MDD might have distinct biological features, despite both conditions showing similarities in depressive behavioral presentations. Clinically, compared to patients with MDD, patients with BD have been found to have earlier and acute onset, and more total episodes [4]. Biologically, evidence from genotypic [5] and endophenotypic [6] studies have shown different biological characteristics between BD and MDD. Neuroimaging studies revealed that compared to MDD, BD patients demonstrate more widespread white matter abnormalities, grey matter volume reductions, and different aberrant functional connectivity in the neural circuitries responsible for emotion regulation, attentional control [7] and reward-processing [8]. Zhao et al. suggested in a meta-analysis abnormal inhibition associated with the cerebello-thalamo-prefrontal circuitry in psychiatric conditions, including MDD and BD [7]. Also, recent functional studies showed that the mania/hypomania symptoms in BD were associated with elevated reward-related activation in dopamine-rich brain regions [8, 9], while blunted striatal signaling might constitute a risk factor for MDD [10]. However, few disorder-specific neuroanatomical features have yet been identified in BD and MDD, and their underlying neurobiological mechanisms remain unclear.

In this study, we aimed to identify these features distinguishing between BD and MDD by adopting the cortical-surface based technique to examine two virtually orthogonal components of cortical volume with distinct genetic determinants [11], namely, cortical thickness and surface area. Cortical thickness has been associated primarily with intermediate progenitor cells [12], which function as neurogenic transient amplifying cells to form ontogenetic columns during cerebral cortical developments. Surface area, on the other hand, has been associated with the proliferation of radial unit progenitors, which consist of neuroepithelial cells and radial glial cells [13], and functionally involve in the mechanisms of arborization, pruning within grey matter [14], and varying myelination at the grey/white matter interface [15]. Given that cortical thickness and surface area have varying genetic influences, investigating them separately would allow understanding of distinct neurobiological mechanisms underlying disorder-specific conditions. To our knowledge, only a few imaging studies have directly compared BD and MDD [16, 17], and most of these have focused on cortical thickness. For instance, Lan and colleagues reported that relative to healthy controls, thinner caudal middle frontal cortex was exclusively found in patients with BD, but not MDD [16]. Other studies examined cortical thickness separately in isolation of the disorders. Han and colleagues examined cortical thickness, together with cortical and subcortical volumes and white matter integrity in patients with first episode of MDD. Cortical volume reductions have been reported in caudal anterior cingulate, caudal frontal gyrus and medial orbitofrontal gyrus in MDD patients [18]. However, in these studies, measures of surface area were not examined. With BD and MDD sharing similar clinical presentations but may have potentially distinct neuroanatomical features, it would be necessary to compare the surface area and the subcortical regions in the dopamine-rich mesocorticolimbic pathways that have been consistently found to be abnormal in patients with BD and MDD [19–22]. These include thalamus, caudate nucleus, putamen, nucleus accumbens, hippocampus and amygdala.

Recently, machine-learning based discriminating classifications have been employed to distinguish between patients with different mental disorders utilizing individual structural and functional brain images. Such sophisticated statistical learning models analyze imaging data by recognizing specific patterns, allowing the distinction of disorders with application to aid diagnostic decisions [23, 24]. For instance, support vector machine-based (SVM) algorithms have been used experimentally to classify patients with Alzheimer’s disease [25–27], autism [28], schizophrenia and depression [29–31]. In a pilot study that studied patients with affective disorders, Grotegerd and colleagues successfully discriminated between patients with unipolar and bipolar depressive status by pattern-classifying their functional images [29]. This neuroimaging-aided approach allows the differentiation of diagnoses based on disorder-specific biological features. Similar but improved automatic classification using multimodal brain structural features is adopted in this study.

In this study, the primary analyses involved whole brain comparisons on cerebral cortical thickness and surface area between patients with BD and MDD, as well as with a group of healthy volunteers in an exploratory manner. We then examined the volumes of subcortical regions-of-interest (ROI), which have been found to be associated with BD and MDD. Based on the ROI morphological features identified from primary analyses, SVM discriminating classification was subsequently computed to distinguish between patients with BD and MDD. Applying parameters of specific cortical and subcortical features would improve the overall accuracy of the discriminating classification. We hypothesized that BD and MDD would have shared but also distinct neuroanatomical features, in terms of cortical thickness, surface area and subcortical volumes especially in the regions involved in the mesocorticolimbic pathways. Multimodal classification on these disorder-specific features would aid the discrimination between BD and MDD.

## Methods

### Participants

A total of 16 patients diagnosed with BD (mixed types of BD-I, BD-IIs, and BD-NOS; 6 males, mean age = 26.3 years, *SD* = 7.9 years) and 19 patients diagnosed with MDD (8 males, mean age = 30.0 years, *SD* = 8.9 years) were recruited from local hospitals in Beijing, China (Institute of Mental Health, The Third Affiliated Hospital of Beijing Chinese Medicine University, and Beijing Hui-Long-Guan Hospital). Patients were diagnosed by experienced psychiatrists (MQ, YTM, ZRW) according to DSM-IV criteria [32]. For comparison purpose, a group of 29 healthy controls (HC, 11 males, mean age = 27.1 years, *SD* = 8.4 years) were also recruited from communities in Beijng. They were demographically (handedness, age, gender, educational level and IQ) well matched (see Table 1). All participants in this study were right-handed, as measured by the Annett Handedness Scale [33]. Their IQ scores were estimated using the short form of the Chinese version of the Wechsler Adult Intelligence Scale-Revised (WAIS-R) [34]. The IQ estimates and handedness were assessed by trained researchers (QZ, ZL). Exclusion criteria for both clinical groups included patients with psychiatric comorbidities [18]; a history of psychotic symptoms; a history of neurological disorder; a lifetime prevalence of substance abuse; an IQ estimate lower than 70; and not being able to be scanned by MRI. Exclusion criteria for healthy controls were similar to the clinical groups, with the addition of no family history of neuropsychiatric or neurological disorders. This study received ethical approvals from the three local hospitals (Institute of Mental Health; The Third Affiliated Hospital of Beijing Chinese Medicine University; Beijing Hui-Long-Guan Hospital) and the Institute of Psychology, Chinese Academy of Sciences. Written informed consent was obtained from each participant before the study.

**Table 1:** Demographic and clinical characteristics

	BD (n = 16)	MDD (n = 19)	HC (n = 29)	F/χ^2^/t-scores	*p*-value
Male % (male/female)	37.5 % (6/10)	42.1 % (8/11)	37.9 % (11/18)	χ^2^ = 0.11	0.95
Right-handed %	100	100	100	F(2) = 0.00	1.00
Age (in years)	26.3 (7.9)	30.0 (8.9)	27.1 (8.4)	F(2) = 1.02	0.37
Education (in years)	15.1 (1.8)	14.4 (3.2)	14.9 (2.5)	F(2) = 0.42	0.66
IQ estimates	116.9 (16.1)	116.7 (13.1)	120.4 (12.7)	F(2) = 0.54	0.59
Intracranial Volume (ICV, cm^3^)	1295 (207)	1439 (195)	1372 (201)	F(2) = 2.24	0.12
Medication History					
Untreated (N)	5	8	/	/	/
Antidepressants (N)	5	11	/	/	/
Antipsychotics (N)	8	2	/	/	/
CPZ (mg)	226.2 (327.4)	38.3 (40.1)	/	/	/
Atypical (N)	8	1	/	/	/
Typical (N)	0	1	/	/	/
Trihexyphenidyl (N)	1	0	/	/	/
Antianxiety (N)	2	3	/	/	/
Lithium (N)	2	0	/	/	/
Valproate (N)	2	0	/	/	/
Lithium + Valproate (N)	1	0	/	/	/
Clinical Information					
Duration of illness (in years)*	5.2 (4.8)	4.9 (3.5)	/	t = -0.23	0.82
HAMD-17*	9.9 (4.5)	11.1 (4.3)	/	t = 0.77	0.45
YMRS*	6.40 (6.9)	2.2 (2.6)	/	t = -2.35	0.03

Abbreviations: *MDD* Major Depressive Disorder, *BD* Bipolar Disorder, *HC* Health Controls, *YMRS* Young Mania Rating Scale, *HAMD-17* Hamilton rating scale for Depression-17 items, *N* number of patients

*p-values were generated from comparisons between the BD and MDD groups (t-tests, two-tailed)

Chi-squared test was used in gender. One-way analyses of variance (ANOVA) were used in age, IQ estimates, handedness, education and ICV

### Medication and clinical characteristics

Medication histories of the two clinical groups are summarized in Table 1. The doses of antipsychotics in medication were transformed into Chlorpromazine equivalent (CPZ) [35, 36]. In the MDD group, eight of 19 patients (42.1 %) were medication-free at the time of assessment. The remaining 11 patients were taking antidepressants, including Citalopram, Mirtazapine, Venlafaxine, Sertraline, Paroxetine, Fluoxetine, and Duloxetine hydrochloride. Among the 11 medicated patients, two of them were also taking antipsychotics (Flupentixol dihydrochloride, CPZ = 10 mg; and Aripiprazole, CPZ = 67 mg); three of them were taking anti-anxiety medicines (including Clonazepam, Buspirone, and Lorazepam). In the BD group, five of 16 patients (31.3 %) were medication-free at the time of assessment. Among the 11 medicated patients, five of them were taking antidepressants (including Venlafaxine, Sertraline, Fluoxetine, Fluvoxamine, Duloxetine hydrochloride); eight of them were taking atypical antipsychotics (including Olanzapine, Quetiapine, Risperidone, Aripiprazole, mean CPZ = 226.2 mg, *SD* = 327.4 mg); one of them was taking Trihexyphenidyl; five of them were taking mood stabilizers (Lithium and Valproate); and two of them were taking anti-anxiety medicines (Clonazepam and Lorazepam).

No group difference was found for duration of illness (*p* >0.05) between patients with BD and MDD. In both clinical groups, major depressive symptoms and manic symptoms were assessed by experienced psychiatrists (MQ, YTM, ZRW) using the 17-item Hamilton Rating Scale for Depression (HAMD) [37] and the Young Mania Rating Scale (YMRS) [38]. No difference was found between patients with BD and MDD in the 17-item HAMD total score (*t* = 0.77, *p* = 0.45). However, as expected, lower YMRS total score (*t* = -2.35, *p* = 0.03) was observed in the MDD group than the BD group.

### MRI acquisitions and preprocessing

High-resolution T1-weighted images from all participants were acquired on a 3-Tesla scanner (Siemens 3 T-Trio A Tim, Erlangen, Germany) at the MRI Center of Beijing 306 Hospital. Before image collection, a pre-scan lasting one minute and 10 s was taken and inspected by clinical radiologists to exclude individuals with structural brain abnormalities. The scanning parameters of the T1-weighted images were as follows: slice thickness = 1 mm, TE = 3.01 ms, TR = 2300 ms, 176 slices in sagittal plane, field of view (FOV) = 256 mm, voxel size = 1 x 1 x 1 mm^3^, bandwidth = 240 Hz/pixel, duration = 6 min 56 s. All participants were asked to close their eyes and remain motionless during data collection.

Cortical reconstruction and subcortical volumetric segmentation were performed using the FreeSurfer imaging analysis suite (v5.1.0, http://surfer.nmr.mgh.harvard.edu/) [39]. Details of this pipeline are fully described on its webpage. Briefly, the T1-weighted images were firstly registered to the Talairach space of each participant’s brain with the skulls stripped. Images were then segmented into white/grey matter (WM/GM) tissues based on local and neighbouring intensities. The cortical surface of each hemisphere was inflated to an average spherical surface to locate both the grey matter (pial) surface and the WM/GM boundary. Preprocessed images were visually inspected (by GF and YD) to ensure the reconstruction and segmentation qualities. Any topological defects were excluded from the subsequent analyses but no data had to be excluded at this point. At the cortical level, cortical thickness was measured as the shortest distance between the pial surface and the GM/WM boundary at each point across the cortical mantle. Surface area was measured as the area of a vertex on the pial surface, calculated as the average of the tessellation areas touching that vertex. In addition, the cerebral cortex of each participant was automatically parcellated into 70 regions according to the Desikan-Killiany cortical atlas [40], with their mean cortical thickness and surface area calculated for the ROI analysis. Before group-level statistical analyses, individual cortical surface maps were smoothed with a Gaussian 25 mm full-width-at-half-maximum (FWHM) kernel when accounting for the sample size. At the subcortical level, volumes of a series of subcortical structures were extracted using the automated segmentation function in FreeSurfer [41] for the ROI analysis. Total intracranial volume (ICV) of each participant was also extracted.

### Vertex-wise group comparison on cortical measures

Whole brain analyses of cortical thickness and surface area were performed pairwise between each two groups of participants (viz., BD vs MDD, HC vs BD, HC vs MDD) using general linear models (GLM) in FreeSurfer’s QDEC (Query, Design, Estimate, Contrast) operation, after co-varying for ICV, age and IQ estimates. In an exploratory manner, the significant threshold was set at *p* < 0.01 uncorrected (two-tailed). To minimize Type I error, only clusters with significant number of vertices greater than 200 were reported [42]. Significant clusters were mapped to the Desikan-Killiany cortical atlas [40] based on the structures of gyrus and sulcus. Group difference maps were constructed in QDEC based on –log_10_ (*p*-value).

### Region-of-Interest (ROI) analyses

ROI analyses were performed between the BD group and the MDD group. At the cortical level, significant clusters from the BD-MDD whole brain comparison would be defined as ROI(s). At the subcortical level, bilateral thalamus, caudate nucleus, putamen, hippocampus, amygdala and nucleus accumbens were defined as ROIs. To control for individual variations of ICV, age and IQ estimate, a ratio of each ROI was calculated [ratio = ROI mean value/(ICV * age * IQ estimate)]. Two sample *t*-tests were performed on the ROI ratios, with the false discovery rate (FDR) corrected for multiple comparisons.

### SVM classification

Using the ROI morphological features as input data, an exploratory SVM was applied to classify patients with BD and patients with MDD. SVM is a supervised multivariate classification algorithm based on pattern recognition. In brief, it separates the input data into different classes (i.e., patients with BD and MDD) by identifying an optimal separating hyperplane (OSH) or named decision boundary. Initially the algorithm is trained on a subset of training data to find a hyperplane that best separates the input data space according to their known class labels (i.e., patients’ group memberships, -1 = MDD and 1 = BD). After the hyperplane is built by a set of support vectors, a subset of test data are then classified using the predicted values to determine which side of the hyperplane they should locate. Given that the input data of the present study are already dimensionally reduced ROI features, a non-linear algorithm was chosen with a radial basis function kernel. Parameters C and gamma, which controls a tradeoff between allowed training errors and misclassifications, and the width of the radial basis function, were tuned using a 10-fold cross-validation approach. The optimized parameters that provide the best accuracy would be selected for the final model.

In the present study, the classifier’s performance is evaluated using the common leave-one-out-each-group cross-validation approach. This validation procedure provides robust parameter estimates particularly for smaller samples [22]. In each trial observation, one patient per group was left out from the data to train the classifier, but then used to determine the detection rate of this trained classifier (testing). The procedure was repeated until every participant had been used for testing a classifier. The overall accuracy of the classifier was the averaged detection rate. The sensitivity and specificity of the classifier were also quantified. Specifically, sensitivity was calculated by the number of true BD dividing by the total number of true BD and those misclassified BD as MDD. Specificity was calculated by the number of true MDD dividing by the total number of true MDD and those misclassified MDD as BD. To evaluate the probability of obtaining the overall accuracy by chance, statistical significance was verified by means of permutation tests [24]. We randomly assigned a class label to each patient and repeated the same cross-validation procedures for 1000 times. Then we counted the total number of times that the detection rates from the permutation tests were higher than or equal to the actual value obtained from the real test. A *p*-value for classification is derived from dividing this number by 1000. The classifications were performed using R version 2.15.3 (R Development Core Team 2013. The R Foundation for Statistical Computing, Vienna, Austria. ISBN 3-900051-07-0, URL http://www.R-project.org), with packages “bootstrap”, “class”, and “e1071” implemented [43].

## Results

### Vertex-wise cortical thickness and surface area

**Fig. 1:**
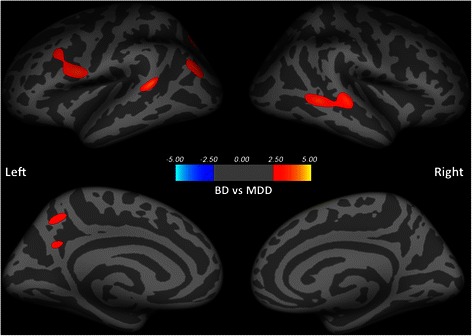
Group differences in surface area between patients with BD and patients with MDD after controlling for age, IQ and the total intracranial volume. Six clusters of larger surface area were observed in patients with BD, when compared to patients with MDD, including the left bankssts, the left precentral gyrus, the left inferioparietal gyrus, the left superiorparietal gyrus, the left precuneus and the right middle temporal gyrus. Larger surface area was colored in red. Clusters were overlaid on the average inflated image. Significance threshold was set to *p* < 0.01 (uncorrected). Clusters with number of significant vertices greater than 200 were displayed. BD: Bipolar Disorder (*n* = 16); MDD: Major Depressive Disorder (*n* = 19)

No group difference in cortical thickness was observed between patients with BD and patients with MDD, after controlling for the possible individual variations in ICV, age and IQ estimate. However, compared to the MDD group, the BD group revealed larger surface area in left bankssts, precentral, inferior parietal, superior parietal, precuneus gyri, and the right middle temporal gyrus (see Table 2 and Fig. 1).

**Table 2:** Significant differences in cortical thickness and surface area between patients with bipolar disorder, patients with major depressive disorder and healthy controls after controlling for age, IQ and total intracranial volume

	Measure		Annotation	log(p)	Size (mm^2)	Talairach coordinates			Number of vertices
						X	Y	Z	
BD > MDD	area.pial	L	bankssts	3.576	224.15	-58.5	-43.8	10.0	458
			precentral	3.138	584.71	-51.4	3.2	18.7	1280
			inferiorparietal	2.812	313.97	-42.2	-72.9	25.6	534
			superiorparietal	2.670	107.18	-20.3	-68.9	35.3	208
			precuneus	2.171	124.20	-10.4	-55.1	44.7	251
		R	middletemporal	3.419	812.33	62.4	-46.4	-0.5	1762
HC > BD	area.pial	L	posteriorcingulate	2.658	175.49	-2.7	-24.4	26.5	486
	thickness	R	caudalanteriorcingulate	2.182	105.74	3.4	9.5	25.8	259
HC < BD	area.pial	L	postcentral	-2.931	195.56	-44.9	-30.0	49.8	494
			precuneus	-2.528	196.41	-20.2	-65.8	24.6	375
			supramarginal	-2.340	160.41	-50.3	-48.5	37.0	374
		R	superiortemporal	-3.884	702.71	44.3	-28.1	-4.7	1641
			insula	-2.684	153.36	39.8	-24.0	-1.9	464
			supramarginal	-2.204	127.56	52.7	-24.5	23.1	307
HC > MDD	thickness	L	medialorbitofrontal	3.008	837.10	-12.5	43.8	0.7	1429
			parsopercularis	2.894	670.07	-37.6	8.3	12.6	1926
			middletemporal	2.504	292.69	-42.4	6.8	-35.0	340
	area.pial	L	rostralmiddlefrontal	2.922	427.44	-39.4	20.9	30.0	832
HC < MDD	area.pial	L	insula	-2.870	170.71	-34.0	-0.2	14.7	482
		R	supramarginal	-2.393	91.08	51.0	-24.6	21.8	215

*p* < 0.01, uncorrected, number of vertices > 200. *L* left hemisphere, *R* right hemisphere. *HC* Healthy Controls (n = 29), *MDD* Major Depressive Disorder (n = 19), *BD* Bipolar Disorder (n = 16)

**Fig. 2:**
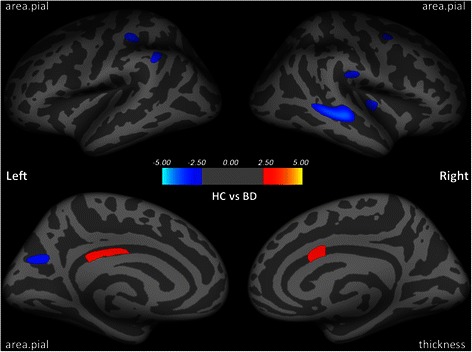
Group differences in cortical thickness and surface area between healthy controls and patients with BD after controlling for age, IQ and the total intracranial volume. Six clusters of smaller surface area were observed in healthy controls, when compared to patients with BD, including the left postcentral, precuneus, supramarginal, the right superiortemporal, insula and supramarginal gyri. Larger surface area of the left posterior cingulate and thicker caudal anterior cingulate cortex were observed in healthy controls. Larger surface area/thickness was colored in red. Smaller surface area/thickness was colored in blue. Clusters were overlaid on the average inflated image. Significance threshold was set to *p* < 0.01 (uncorrected). Clusters with number of significant vertices greater than 200 were displayed. BD: Bipolar Disorder (*n* = 16); HC: Healthy Control (*n* = 29)

Compared to the HC group, patients with BD had thinner caudal anterior cingulate cortex (cACC) in the right hemisphere, and reduced surface area in the left posterior cingulate cortex (PCC). In contrast, patients with BD had larger surface area than healthy controls in the left postcentral, precuneus, supramarginal gyri, and in the right superior temporal, insula and supramarginal gyri (see Table 2 and Fig. 2).

**Fig. 3:**
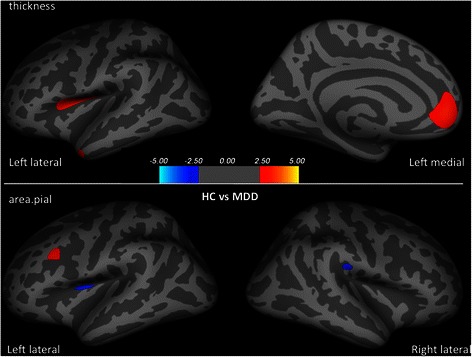
Group differences in cortical thickness and surface area between healthy controls and patients with MDD after controlling for age, IQ and the total intracranial volume. Three clusters of larger cortical thickness were observed in healthy controls than patients with MDD, including the left medial orbitofrontal, parsopercularis and middle temporal gyri. When compared to patients with MDD, healthy controls had larger surface area in the left rostral middle frontal gyrus, but smaller surface area in the left insula and the right supramaginal gyrus. Larger surface area/thickness was colored in red. Smaller surface area/thickness was colored in blue. Clusters were overlaid on the average inflated image. Significance threshold was set to *p* < 0.01 (uncorrected). Clusters with number of significant vertices greater than 200 were displayed. MDD: Major Depressive Disorder (*n* = 19); HC: Healthy Control (*n* = 29)

Compared to the HC group, patients with MDD had thinner cortices in the left medial orbito-frontal, parsopercularis, middle temporal gyri, and reduced surface area in the left rostral middle frontal gyrus. Larger surface area in the left insula and the right supramarginal gyrus were found in patients with MDD when compared to HCs (see Table 2 and Fig. 3).

### ROI results

Six ROIs were defined from the BD-MDD whole brain group comparison at the cortical level. Similar to the vertex-by-vertex comparison findings, the BD group had larger surface areas than the MDD group in the left superior parietal, left precuneus and the right middle temporal gyri (*p* = 0.05, FDR corrected). Similar trends were also found in the other three ROIs, despite not reaching statistical significance (*p* < 0.1, FDR corrected, see Table 3).

**Table 3:** Differences in regions-of-interest (ROIs) between patients with bipolar disorder and patients with major depressive disorder after controlling for age, IQ and total intracranial volume

	ROI	df	Mean in BD	Mean in MDD	t	*p*	*p*.adj (FDR)
Cortical (area.pial)	L bankssts	32.09	2.80E-07	2.31E-07	1.73	0.09	0.09
	L precentral	29.01	1.33E-06	1.11E-06	1.83	0.08	0.09
	L inferiorparietal	32.28	1.32E-06	1.09E-06	1.91	0.07	0.09
	L superiorparietal	28.98	1.53E-06	1.17E-06	2.45	0.02	0.05
	L precuneus	28.98	1.10E-06	8.56E-07	2.41	0.02	0.05
	R middletemporal	27.53	9.76E-07	7.59E-07	2.43	0.02	0.05
Subcortical (volume)	L thalamus	25.72	1.99E-06	1.60E-06	1.95	0.06	0.08
	L caudate nucleus	28.90	9.85E-07	8.13E-07	1.84	0.08	0.08
	L putamen	26.15	1.70E-06	1.36E-06	1.99	0.06	0.08
	L hippocampus	23.37	1.20E-06	9.15E-07	2.42	0.02	0.05
	L amygdala	25.70	4.95E-07	3.83E-07	2.68	0.01	0.05
	L nucleus accumbens	25.27	1.64E-07	1.17E-07	2.52	0.02	0.05
	R thalamus	25.07	1.94E-06	1.60E-06	1.89	0.07	0.08
	R caudate nucleus	27.58	1.01E-06	8.17E-07	1.89	0.07	0.08
	R putamen	26.37	1.65E-06	1.31E-06	2.07	0.05	0.08
	R hippocampus	24.94	1.24E-06	9.56E-07	2.57	0.02	0.05
	R amygdala	27.23	5.03E-07	3.96E-07	2.44	0.02	0.05
	R nucleus accumbens	28.10	1.84E-07	1.36E-07	2.47	0.02	0.05

A ratio of each ROI was calculated to control for the individual variations of ICV, age and IQ. *Ratio* ROI mean value/(ICV * age * IQ estimate), *df* degree of freedom, *p.adj (FDR)* adjusted *p*-value, with false discovery rate corrected. *BD* Bipolar Disorder (n = 16), *MDD* Major Depressive Disorder (n = 19)

At the subcortical level, the BD group was found to have larger volumes in the ROIs than the MDD group, especially in the bilateral hippocampus, amygdala and nucleus accumbens (*p* = 0.05, FDR corrected, see Table 3).

### SVM classification results

**Fig. 4:**
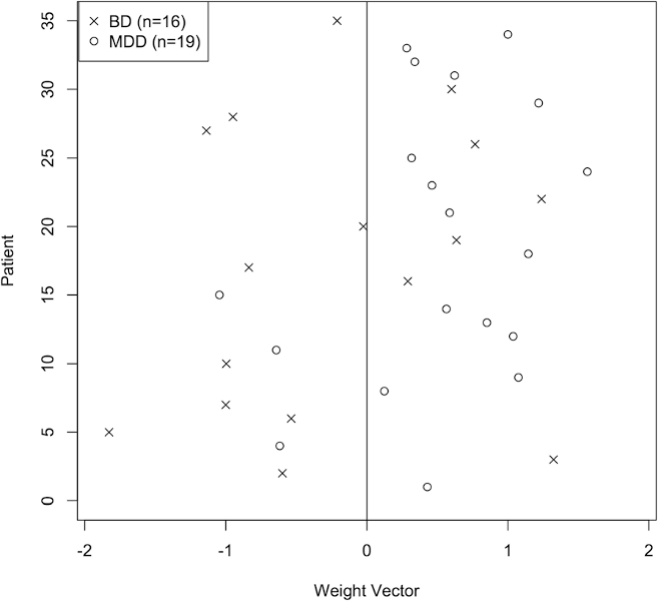
Classification plots showing group allocation of patients with BD and MDD

Eighteen ROIs (i.e., 6 cortical and 12 subcortical regions) listed in Table 3 were initially defined as discriminating morphological features to classify between patients with BD and MDD. Their ratio values were used in the analyses to control for the individual variations of ICV, age and IQ estimate. The optimal parameters for the radial basis function kernel were set to C = 100 and gamma = 0.0001 after tuning. In the final model, the overall accuracy of discriminating between patients with BD and MDD was 74.3 %, with a sensitivity of 62.5 % and a specificity of 84.2 % (*p* = 0.028). This means that 26 out of 35 patients were correctly classified into the accurate diagnostic category after taking into account the probability of randomness (see Fig. 4). Moreover, 10 out of 16 BD patients were correctly classified, with the other 6 misclassified as MDD patients (sensitivity), and 16 out of 19 MDD patients were successfully classified, with the other 3 misclassified as BD patients (specificity).

Similar results were obtained, when we repeated the analyses using only the ROIs that were found to be significantly different between patients with BD and MDD (i.e., 3 cortical and 6 subcortical ROIs in Table 3). With these 9 ROIs as input features, the overall accuracy was 71.4 %, with a sensitivity of 56.3 % and a specificity of 84.2 % (*p* = 0.019, C = 100, gamma = 0.0001).

## Discussion

We found significant differences in surface area and subcortical volumes between BD and MDD, with application to neuroanatomical classification between these two conditions with adequate accuracy. Identifying disorder-specific biological features of BD and MDD has important clinical implications in facilitating efficient and specific treatments. Given that BD and MDD share similar depressive symptoms, patients with BD who have not yet developed a history of mania frequently got misdiagnosed as MDD [17]. Moreover, inadequate treatments in patients with BD may increase the risk of rapid cycling between mood states [44]. Therefore, studying disorder-specific biological features that reflect brain developmental pathways and mechanisms, such as cortical thickness and surface area, would aid the development of reliable diagnostic tools to distinguish between BD and MDD.

Larger surface area was observed in patients with BD in comparison to MDD in the left fronto-temporo-parietal regions, as well as the right temporal lobe. However, no significant difference in cortical thickness was observed between BD and MDD. This is in contrast to a recent study reporting that patients with BD had thinner cortices in the left inferior parietal gyrus, the right caudal middle frontal gyrus and precuneus when compared to patients with MDD [16]. This discrepancy could be due to variations in intracranial volumes, which was carefully controlled for in this study. Furthermore, compared to HC, patients with BD had larger surface area in the temporo-parietal regions, but thinner cortices and reduced surface area in the cingulate cortices in both hemispheres. However, patients with MDD revealed significant reductions of cortical thickness in the left medial orbitofrontal and parsopercularis gyri, when compared to healthy controls. Despite certain discrepancies are likely to be attributable to the heterogeneity of these disorders, our findings are in line with other volumetric studies showing that both conditions have abnormalities in the anterior cingulate cortex, dorsolateral prefrontal cortex and orbitofrontal cortex [19, 45]. In patients with BD, reduced grey matter volumes in the prefrontal and temporal lobes have been consistently reported, particularly in the orbitofrontal gyrus, a region found to have functional impact in affective processing and decision making [46]. However, studies of grey matter volume conflate the contributions of cortical thickness and surface area as each of these two dimensions provide important neurobiological information on the basic structural elements in brain development. Therefore, by delineating the measurements of volume into cortical thickness and surface area, our findings aid the understanding of underlying neuropathological processes of the cerebral cortex in patients with BD and MDD.

In cortical development, neurons within the cerebral cortex are organized into ontogenetic columns [47]. According to the radial unit hypothesis [48], cells within a column share a common origin and migrate to their location within the cortex. Cortical thickness, which contains the intermediate progenitor cells, reflects the number of neurons produced within each column. However, the size of surface area reflecting proliferation of radial unit progenitor cells is driven by the number of ontogenetic columns [13]. In this study, reductions of cortical thickness were found in the left medial orbitofrontal cortex and parsopercularis (Brodmann area 44) in patients with MDD as compared to healthy controls, indicating a possibility of neuron number reductions in these regions. Orbitofrontal cortex and parsopercularis of the inferior frontal gyrus are brain regions involved in emotional processing. Brain volume reduction in these areas has been evidenced as one of characteristics in MDD [49] and abnormalities in the orbitofrontal regions were observed to be cytoarchitectionically distinct with functional consequence with respect to mood regulation [50]. Similar to cortical thickness, surface area is highly heritable [11], yet has received less attention in human imaging research. In this study, larger surface area was found in patients with BD relative to MDD and healthy controls. According to the tension-based theory of morphogenesis [51], it is speculated that surface area expansion might be indirectly associated with white matter tracts damage, as more tension from white matter fibers shrinkage could lead to deeper sulci and extended cortical surface area. Therefore, the expansion of surface area observed in our BD sample may suggest damages of white matter tracts in the temporo-parietal regions, which deserves further investigation using diffusion tensor images. This speculation is supported by a recent meta-analytical study showing that relative to patients with MDD, BD patients had a greater reduction in fractional anisotropy (FA) in the left posterior cingulum [52]. Taken together, our findings suggest that volumetric alterations reported in BD patients [18] might be the result of variations in the surface area, while cortical thickness may play a more important role in MDD patients [18, 19, 53]. Variations of brain volume in healthy adults [54] and other psychiatric conditions, such as autism [12, 55], are driven predominately by differences in cortical thickness or surface area. Variability in cortical thickness and surface area in patients with BD and MDD may thus reflect different underlying neuropathological underpinnings in the development of the disorders.

Intriguingly, larger volumes of subcortical regions involved in the mesocorticolimbic pathways were found in patients with BD as compared to MDD, after controlling for age, IQ estimate and intracranial volumes. When compared to healthy controls, patients with MDD revealed reduced volumes in the caudate, nucleus accumbens, hippocampus and amygdala [49]. Reduced neural responses to rewards in these regions have also been reported [56]. However, in contrast to the blunted striatal signaling in MDD, elevated ventral striatal, orbitofrontal and ventrolateral prefrontal activities during reward anticipation have been observed in patients with BD [8, 9]. Therefore, the larger volumes of these dopamine-rich regions we found in BD patients may provide additional evidence to the emerging reward hypersensitivity theory in BD.

By applying SVM discriminating classification on these ROI morphological features, we were able to distinguish between BD and MDD patients with up to 74.3 % correct classification rate. Different from the whole brain classification approaches [29–31], in the present study, we subsequently reduced the feature parameters by selecting a subset of ROIs that best distinguished between BD and MDD. In a relatively small sample, this noise-reduction approach allows loading high weights on the significant features (i.e., ROIs), resulting in an increase of diagnostic accuracy. It is of note that, in our sample, six patients with BD were misclassified as MDD (sensitivity, 62.5 %) and three patients with MDD were misclassified as BD (specificity, 84.3 %). This pattern is in line with recent findings [57, 58] and the clinical complexities in diagnosing BD from MDD. Patients with onset of depressive episodes are often diagnosed and treated as having MDD until a manic or hypomanic episode emerges. The consequence of misdiagnosis could lead to poor clinical outcomes resulting from ineffective pharmacological treatments and delaying the course of illness. More importantly, studies have reported an association between antidepressants and an elevated risk of hypomanic, mania and rapid cycling [59]. The treatments for MDD in BD patients may thus likely induce the risk and frequency of manic/hypomanic episodes. Therefore, accurately diagnosing BD and MDD has significant therapeutic and prognostic importance. Current clinical practice that relies solely on symptomatology in making diagnosis renders relatively low sensitivity (BD: 45 %; MDD: 40 %) and specificity (BD: 81 %; MDD: 87 %) [60, 61]. The application of imaging-aided classification approach may therefore help to improve accuracy in diagnostic decisions for targeted treatment.

We acknowledged three limitations in this study. Firstly, the possible medication effects on brain morphological alterations could not be estimated in this study. The neurotropic effect of antidepressants on brain anatomical changes has previously been reported [62]. In addition, multiple recurrent episodes and chronicity of conditions in BD and MDD may impact on the observed morphometric abnormalities. Further investigations on these disorder-related parameters, such as medication and the number of recurrent episodes, are therefore warranted. Secondly, the subtypes of BD were not examined separately in this study due to the small sample size. BD-II is generally considered as a milder form of manic-depressive disorder from its less severe symptom intensity, yet it was reported to be more severe in terms of its episode frequency [63]. The clinical differences between BD-I, BD-II and BD-NOS may likely suggest potential biological differences. Thirdly, the relatively small sample size may potentially lend to Type I errors in the SVM classification. Therefore, specific patterns we reported must be considered as preliminary. Applying machine learning techniques to replicate neuroanatomical models across samples in a larger scale is warranted. In addition, in view of the explorative nature of this study, permutation statistical control for multiple comparisons was not carried out for the initial whole brain analyses (see similar [45]). The resulting clusters from the whole brain analyses in this study are considered robust as indicative by the large number of significant vertices. Moreover, the clusters defined as ROIs for subsequent analyses reached a stringent significant threshold of 0.001, approximately equivalent to a *p* < 0.05 corrected for multiple comparisons, when *a priori* hypothesis was present [64]. Taken together, replication studies to examine cortical thickness and surface area by separating subtypes of BD in a larger sample would be beneficial to elucidate the underlying neurobiological differences between BD and MDD, which is a part of our on-going work.

## Conclusions

We observed distinct spatially distributed variations in cortical thickness and surface area in patients with BD and MDD. Potentially, these findings may reflect neuropathological processes of the two disorders, implying that the two disorders may vary etiologically. Our finding supports separating the two disorders into different clinical entities, despite some similar affective symptoms are shared in common. Finally, this study has shed light to the adoption of a multimodal classification on disorder-specific biological features to aid diagnostic decisions with significant therapeutic and prognostic implications.

## Abbreviations

BD: Bipolar disorder
CPZ: Chlorpromazine equivalence
DSM-5: Diagnostic and statistical manual of mental disorders
FDR: False discovery rate
FOV: Field of view
FWHM: Full-width-at-half-maximum
GM: Grey matter
HAMD: Hamilton Rating Scale for Depression
HC: Healthy controls
ICV: Intracranial volume
IQ: Intelligence quotient
MDD: Major depressive disorder
MRI: Magnetic resonance imaging
OSH: Optimal separating hyperplane
QDEC: Query, Design, Estimate, Contrast
ROI: Regions of interest
SD: Standard deviation
SVM: Support Vector Machine
WAIS-R: Wechsler Adult Intelligence Scale-Revised
WM: White matter
YMRS: Young Mania Rating Scale
